# The direct perception hypothesis: perceiving the intention of another’s action hinders its precise imitation

**DOI:** 10.3389/fpsyg.2014.00065

**Published:** 2014-02-18

**Authors:** Tom Froese, David A. Leavens

**Affiliations:** ^1^Departamento de Ciencias de la Computación, Instituto de Investigaciones en Matemáticas Aplicadas y en Sistemas, Universidad Nacional Autónoma de MéxicoMexico City, Mexico; ^2^Centro de Ciencias de la Complejidad, Universidad Nacional Autónoma de MéxicoMexico City, Mexico; ^3^School of Psychology, University of SussexBrighton, UK

**Keywords:** phenomenology, perception, symbolic culture, development, chimpanzee, social cognition, enculturation, comparative psychology

## Abstract

We argue that imitation is a learning response to unintelligible actions, especially to social conventions. Various strands of evidence are converging on this conclusion, but further progress has been hampered by an outdated theory of perceptual experience. Comparative psychology continues to be premised on the doctrine that humans and non-human primates only perceive others’ physical “surface behavior,” while mental states are perceptually inaccessible. However, a growing consensus in social cognition research accepts the direct perception hypothesis: primarily we *see* what others aim to do; we do not infer it from their motions. Indeed, physical details are overlooked – unless the action is unintelligible. On this basis we hypothesize that apes’ propensity to copy the goal of an action, rather than its precise means, is largely dependent on its perceived intelligibility. Conversely, children copy means more often than adults and apes because, uniquely, much adult human behavior is completely unintelligible to unenculturated observers due to the pervasiveness of arbitrary social conventions, as exemplified by customs, rituals, and languages. We expect the propensity to imitate to be inversely correlated with the familiarity of cultural practices, as indexed by age and/or socio-cultural competence. The direct perception hypothesis thereby helps to parsimoniously explain the most important findings of imitation research, including children’s over-imitation and other species-typical and age-related variations.

## INTRODUCTION

Theories in developmental and comparative psychology have been undergoing drastic changes, mainly driven by unexpected experimental findings. Not too long ago most primatologists believed that non-human primates, including our closest relatives the chimpanzees, lacked the capacity of understanding conspecifics as other intentional agents like themselves (Tomasello, 1999). But more recent experimental designs are revealing more of the actual extent of their social understanding (Call and Tomasello, 2008). Similarly, there has been a trend in developmental psychology to demonstrate various aspects of social cognition in increasingly younger human infants by using non-verbal, behavior-based experimental paradigms, thereby contradicting a long-held theory that “theory of mind” first emerges around 4 years of age (Baillargeon et al., 2010). These discoveries have stimulated an ongoing discussion about which theories can account for these new developmental and comparative data (Hutto et al., 2011). Following Gallagher, we agree that they best fit with recent theoretical developments in social cognition research that is focused on active perception, embodied cognition, and phenomenology (Gallagher and Povinelli, 2012). We contribute to these changes by critically evaluating current theories of imitation in comparative psychology.

Imitation is one type of social learning in which both the form and goal of a modeled action is acquired by an agent from another social being (e.g., Whiten and Ham, 1992; Hoppitt and Laland, 2008). Examples of social learning are widespread among animals: for instance, young vervet monkeys will emit “eagle” alarm calls to almost any airborne object, including falling leaves, and with development the response is gradually tuned to airborne predators only (Seyfarth and Cheney, 1986). There are a number of ways in which one organism can influence the probability of another organism displaying a response. One animal, for example, might be foraging in a particular location, which draws the attention of another animal to that location (local enhancement). The mere presence of a conspecific might trigger certain responses; for instance, the probability of re-caching food by scrub jays and ravens increases in the presence of a conspecific observer (Emery and Clayton, 2001; Bugnyar and Heinrich, 2005). In this article, we are concerned with imitation; in particular, how the saliency of the observed action’s goal versus the saliency of its physical means influences the fidelity of the imitative response (for a general review of social learning see, e.g., Hoppitt and Laland, 2008). Although imitation has been explored in a wide variety of animal species, including dogs (e.g., Fugazza and Miklósi, 2013), rats (e.g., Heyes et al., 1992), and pigeons (e.g., McGregor et al., 2006), due to space limitations here we will focus primarily on research involving non-human and human primates.

### DEVELOPMENTS IN IMITATION RESEARCH

The specific unintelligibility of conventional practices, such as customs and language, has been emphasized decades ago (Tomasello et al., 1993) and continues to be noted (Gergely and Csibra, 2006). But research into imitation has so far failed to directly relate such differences in the intelligibility of actions to qualitative differences in the experience of observing them. Instead, it is claimed that all actions are equally perceived as nothing but physical motions, thus requiring mentalistic inferences about their intentions, with some actions being more *cognitively *opaque than others (Csibra and Gergely, 2006). We argue that this neglect of how different categories of action are actually experienced, rather than intellectually assessed, has long resulted in an overemphasis of the role of inferential reasoning in imitation and of imitation’s role in copying instrumental actions. Many studies have investigated variations in the fidelity of copying tool-based actions while neglecting imitation’s role in communicative and other social practices. But this is starting to change on the basis of recent findings in comparative and developmental psychology.

Surprisingly, it was found that children frequently imitate instrumental actions even if they are clearly causally unnecessary to achieve the goal of the demonstrator, thus exhibiting so-called *over-copying *(Whiten et al., 2005b) or *over-imitation *(Lyons et al., 2007)*. *For example, one 3-year-old child twisted a non-functional pin 161 times after seeing a demonstrator twist the same pin only 16 times, in a study by Whiten et al. (1996). Over-imitation has been consistently documented for children, but not for young and older chimpanzees (Nagell et al., 1993; Horner and Whiten, 2005; Whiten et al., 2005b; but see Hobaiter and Byrne, 2010), it is more consistent in 5-year-old than in 3-year-old children (McGuigan et al., 2007), and it has been demonstrated in a cross-cultural context (Nielsen and Tomaselli, 2010). Children can correctly identify the irrelevant actions, for example as being “silly”; they do not copy them merely to please the demonstrator, and they will even continue copying them despite explicit instructions to the contrary (Lyons et al., 2007).

Given these puzzling findings it may seem that human infants develop “towards more ‘mindless’ blanket copying” (Whiten et al., 2009, p. 2427). However, there is a growing consensus that over-imitation is actually a rational learning strategy of a specific class of behaviors. While attempts to relate this phenomenon to causal learning of complex tool-use persist (Lyons et al., 2011), there is increasing evidence that its main purpose is the acquisition of behavioral norms that are based on arbitrary social conventions. Children give conventional explanations of their over-imitated actions, “implying that what they did was prescribed (e.g., ‘I had to do it how they showed me’ or ‘I had to do it the way they did it’)” (Herrmann et al., 2013, pp. 540–541). They protest if others fail to over-imitate (Keupp et al., 2013), and they will continue protesting even after they have seen others succeed at realizing the same goal while omitting the causally unnecessary action (Kenward, 2013).

Accordingly, even researchers who have extensively argued that imitation’s primary function is to enable children’s social learning of complex instrumental actions (e.g., Csibra and Gergely, 2006) are forced to modify their theories to incorporate a more significant role of normativity in tool-use (Király et al., 2013). Nevertheless, we argue that this continuing focus on causal learning of cultural artifacts is a bias derived from our own modern science- and technology-saturated cultural environment. For most of hominid evolutionary history, others’ intentions of tool-use and tool-creation probably were relatively self-evident to conspecifics, especially because for around two million years technological development proceeded at the pace of biological evolution itself (Ambrose, 2001). 

Even unfamiliar instrumental actions can largely be understood in a contextually constrained manner due to the causal necessity of using certain actions to achieve some goal, given the circumstances.

Unfamiliar symbolic actions, on the other hand, tend to be utterly opaque because their underlying means are not determined by causal necessity, but by historically contingent social norms. The conventionally constrained relationship of signifier and signified can be completely arbitrary and therefore must by necessity be acquired by “blind” imitation or pedagogy. To be sure, advanced technologies can reach similar levels of opacity, but when such techniques first developed in the Middle Stone Age, for example the manufacture of compound adhesives, the prerequisites of symbolic cognition were likely already in place (Wadley, 2011). It is therefore possible that it was an increase in social norms in early hominid societies, which first necessitated an improved capacity for faithful imitation, while the improved transmission of advanced instrumental techniques was a beneficial side-effect. We will return to the question of the origins of human imitation at the end of this article.

The crucial qualitative differences between perceiving contextually constrained and conventionally constrained actions have long been ignored because, as we will argue in more detail below, the theory of perception standardly employed in comparative psychology is misguided. Phenomenologists, on the other hand, have begun to remind scientists that the intentions of most observed actions, including instrumental actions, are *directly perceivable* by others – without the necessity of having to overcome any kind of opacity by engaging in mentalistic or behaviorist inference (Gallagher and Povinelli, 2012). This direct perception of intention or meaning makes the perception of the underlying physical details difficult – unless the other’s action is unintelligible to the observer, for example because it is an utterance in an unfamiliar language. This basic phenomenological insight, supported by a variety of psychological evidence that is reviewed further below, has important theoretical consequences for comparative psychology.

It has been widely recognized that humans are cultural animals and that it is adaptive for children to become enculturated as fast as possible, but it has remained puzzling how they can learn general norms from “single observations of tokens of the action” (Rakoczy and Schmidt, 2013, p. 20), especially because it seems that “unfortunately for children, information available in the environment does not come tagged as being cultural” (Diesendruck and Markson, 2011, p. 189). This puzzle has led to an emphasis of the role of ostensive cues and pedagogy in human imitation (Gergely and Csibra, 2006; Király et al., 2013). However, although others can provide guidance, this help is not required for one-shot learning of norms. Children will interpret one unnecessary action as conventionally constrained as long as it is performed intentionally (Schmidt et al., 2011). This ability is less surprising from the phenomenological perspective because novel conventionally constrained actions are indeed to some extent “tagged” as such when perceived by an observer to whom they are unfamiliar. They are directly perceived as intentional actions, yet are simultaneously seen as causally unnecessary and unintelligible. Importantly, this unintelligibility also facilitates faithful imitation because perception of an action’s meaning and perception of its physical means are co-dependent processes, which mutually exclude each other from focus, similar to the relationship between figure and ground. Lack of perceived meaning therefore makes the underlying physical means more salient. Of course, not every intentional action that a child perceives to be non-sense in this way is an unfamiliar norm-governed action, so there will be false positives, especially in artificial experimental situations – precisely what has become known as over-imitation.

In summary, we suggest that children’s over-imitation is a highly selective action by which they specifically pick out those adult actions whose meanings are perceptually unintelligible, and which are therefore most likely determined by social convention. The phenomenological claim that this unintelligibility is manifested as a *perceptual *opacity, rather than as a *cognitive* opacity (Gergely and Csibra, 2006), is supported by a variety of evidence, including the fact that rational imitation can be affected by modulating the perceptual salience of the observed action (Beisert et al., 2012). More generally, it has been repeatedly demonstrated that how we understand others’ actions modulates our perceptual experience of the underlying physical details (Teufel et al., 2010). This and related evidence is discussed in more detail further below. We therefore propose that children’s over-imitation is best understood as a special instance of a more general inverse correlation between an individual’s propensity to imitate an action and that action’s perceptual intelligibility. We suggest that this general inverse correlation is found across primates, but that humans have become adapted to take advantage of it in the service of more effective enculturation during their development.

### DEVELOPMENTS IN SOCIAL COGNITION RESEARCH

Theoretical developments in our understanding of social cognition provide new perspectives for explaining discoveries in comparative and developmental psychology (Racine and Carpendale, 2007; Gallagher and Povinelli, 2012). There are at least two important developments. First, there is the hypothesis of embodied cognition (HEC), which proposes that cognition is primarily embodied and interactive, such that real-time bodily interactions between two or more people can be partially constitutive of some social cognitive processes (see, e.g., De Jaegher and Di Paolo, 2007; Krueger, 2011; Froese and Gallagher, 2012). Second, there is the hypothesis of direct perception (HDP), which proposes that perceptual experience primarily is a process of directly revealing or disclosing the meaning of the perceived (Gallagher, 2008a; Zahavi, 2011). There are two complementary aspects to the HDP.

On the one hand, the HDP implies that when we perceive a part of our physical environment, we directly perceive the meaning it has for us. For example, we perceive an object in terms of its implicit affordances for interaction (Noë, 2004), and these affordances are shaped by our social context including norms of usage (Gallagher, 2008b). On the other hand, the HDP makes a specific claim about how we perceive other people, namely as other agents with mental lives like ourselves. Their bodily presence is encountered as an affordance for social interaction (Krueger, 2012). Moreover, biologically constrained bodily expressions, contextually constrained tool-use, and familiar conventionally constrained practices are directly perceived as intentional and goal-directed. Not all intentions are perceptually transparent to the same degree, and some actions can be deceptive, but in everyday social encounters our direct perceptual insight is often sufficient such that reflection about the other’s beliefs and desires is not necessary for successful social interaction (Ratcliffe, 2007).

Even though the HEC and the HDP can be defended independently, they make good theoretical complements. If the HEC is correct that aspects of social cognition can sometimes be directly realized in embodied social interaction with others, and more generally that cognition can be directly constituted by our embodied comportment in the world, then the HDP becomes less mysterious. The mental lives of others are perceptually accessible because their minds are not hidden inside their brains but embodied and realized in their actions. Other people’s minds are seen in their worldly comportment (Krueger, 2012), and are experienced during social interaction (De Jaegher, 2009). This is especially true of basic emotions (Stout, 2012), but it can also hold for aspects of the classic belief-desire psychology (e.g., I directly perceive that a pupil believes she has failed the exam in her withdrawn expression and slumped posture – no additional explicit thought process is required on my part)^1^.

### OVERVIEW OF THE ARGUMENT

The rest of this article unfolds in four stages. First, we critically examine the theory of perception that has traditionally informed comparative and developmental psychology and show that its logical consequences do not easily fit with the empirical findings of current imitation research. Then we briefly review evidence from phenomenology and psychology to independently motivate the acceptance of a more adequate theory of perception, specifically the HDP. We then argue that the logical consequences of this hypothesis fit better with what is generally known about imitation, and apply the hypothesis to clarify central issues in the debate about the development and evolution of imitation.

## LOGICAL CONSEQUENCES OF THE STANDARD THEORY OF PERCEPTION FOR IMITATION

Cognitive science has traditionally treated perception as a separate input stage that is independent of the rest of the cognitive system. Perception is a form of information processing that converts external physical stimuli into internal mental representations (a transductive process) to be used by the cognitive system for reasoning about the current state of the world and hence what to do next. According to this view –we will call it the hypothesis of physical perception (HPP) – cognition is entirely contained within the transductive envelope of perception, and perception primarily provides agents with a detailed set of facts about the external environment as it is conceived of by classical physics (e.g., the position, volume, velocity, etc., of distinct objects). If the HPP is taken as the theoretical starting point we end up with the following logical deduction about social cognition (Froese et al., 2012a):

[HPP-(1):] The intentional actions of other agents can only be perceived as abstract physical motions in objective space.
[HPP-(2):] Given HPP-(1), the other’s agency and intentions are not directly observed via perceptual experience^2^.
[HPP-(3):] Given HPP-(2), the other’s agency and intentions are secondarily derived and attributed to the observed physical motions by means of additional social cognition (e.g., a “theory of mind mechanism”).

The starting premise of this deduction, HPP-(1), is typically phrased in the literature in terms of the metaphorical contrast between a surface and its hidden content. For example, it was once hotly debated whether chimpanzees can understand that conspecifics have minds of their own, given that perception can only provide access to “surface-level behavior,” and given that this social ability would require them to somehow go “beneath the surface” (Call and Tomasello, 2008, p. 187). Since it is assumed from the beginning that perception cannot do this job (HPP-2), but there is evidence of social understanding in chimpanzees, it is necessary to postulate another cognitive process (HPP-3). A similar process of reasoning is often applied to the social understanding of human children and adults. It follows that HPP-(1) is foundational to the concept of “theory of mind” in comparative and developmental psychology (Froese et al., 2012a). We find explicit claims to this effect by leading experts throughout the whole history of the cognitive sciences.

In saying that an individual has a theory of mind, we mean that the individual imputes mental states to himself and to others (either to conspecifics or to other species as well). A system of inferences of this kind is properly viewed as a theory, first, because *such states are not directly observable*, and second, because the system can be used to make predictions, specifically about the behavior of other organisms. (Premack and Woodruff, 1978, p. 515; emphasis added)

normal children give elaborate verbal descriptions of the *unobservable psychological states* of people, indicating that they relate observable actions to underlying mental states. (Meltzoff, 1995, p. 838; emphasis added)

Generally, the observable behavior of individuals is never transparent either in respect to the background knowledge that governs their actions or in respect to the ultimate goal of the action (if it were transparent, cognitive psychology would not exist as a scientific discipline; Csibra and Gergely, 2006, p. 252).

This classic dualism between bodily behaviors and mental states continues to inflect and bias the debate in comparative psychology (Racine and Carpendale, 2007). For example, Call and Tomasello (2008, p. 189) concluded that “chimpanzees, like humans, understand the actions of others not just in terms of *surface behaviors* but also in terms of the *underlying goals*, and possibly intentions, involved” (emphasis added). In accordance with the HPP, it is claimed that “the goals and perceptions of others are *not readily observable*, and so require inferences” (Tomasello, 2008, p. 176; emphasis added). The possibility that social understanding is a direct perceptual achievement in most normal situations is thereby excluded by definition.

This limited view of perceptual experience has important implications for how researchers in developmental and comparative psychology approach the phenomenon of imitation. We can deduce a couple of predictions about what would happen when an agent, who is operating according to the principles of the HPP, intends to replicate the observed behavior of another agent.

[HPP-(4):] Given HPP-(2), an agent’s replication of observed behavior is primarily guided by the other’s abstract physical motions in objective space and its causal consequences on the environment.
[HPP-(5):] Given HPP-(3), an agent’s replication of observed behavior can also be guided by the other’s intention, but only to the extent that the observer has the additionally required social cognitive capacity.

The ways in which the replication of an observed behavior is guided (with or without goal understanding) and performed (copying means or ends) have been differentiated in the literature. In the absence of any goal understanding, a replication of the means of an observed action is typically referred to as mere “mimicry,” whereas a replication of the means that is also guided by an understanding of the other’s goal is called “imitation” (Tomasello et al., 1993). Nevertheless, this terminology is not consistently applied in the literature since the phenomena of neonatal imitation and children’s over-imitation arguably do not involve an understanding of the other’s goals, and should therefore be classed as forms of mimicry.

A replication of the effects of an action, but by other means than those observed, is not called imitation but “emulation.” The role of goal understanding in emulation is controversial (Huang and Charman, 2005). It was initially proposed that emulation lacks goal understanding, since the replication of the results could be based on the observed results alone (Tomasello et al., 1993). But evidence demonstrating that 18-month-old children re-enact and complete the goals of incomplete or failed actions suggests they employ goal emulation (Meltzoff, 1995), as does evidence that 14-months-olds and enculturated chimpanzees emulate more often when the reasons for the movements are clear to them (Gergely et al., 2002; Buttelmann et al., 2007).

Thus, both the replication of means and ends may involve (and not involve) goal understanding. According to the HPP, the physical means and the physical outcomes of an observed action are both given in perceptual experience, thus seemingly making imitation easy. On the other hand, the means used to emulate an observed result are by definition different from the means of the perceived action (otherwise it would be imitation), thereby requiring a creative response so as to avoid imitation. Accordingly, it seems that the received theory, which holds that imitation is comparatively rarer because it is more complex than emulation, is problematic (Call and Carpenter, 2002). Indeed, following the logic of the HPP, we end up with precisely the opposite conclusion, namely that exact copying of means is less complex and should therefore be the more common form of replication. Mimicry of observed actions is always possible without additional physical or social cognition (i.e., reasoning about causal relations or goals), whereas emulation always requires additional physical cognition to devise alternative means – with or without social cognition (although goal understanding would certainly help to inform the creative process). The HPP-based theories thereby arrive at a puzzling prediction:

[HPP-(6):] Given HPP-(4) and HPP-(5), copying the means of an observed action is cognitively *less* demanding than emulating its intended results.

This is an odd prediction because extensive research in comparative psychology tells us that precisely the opposite should be the case. Faithful imitation is a much less common skill than emulation – some have even argued that it is limited to humans (Tomasello, 2008). But if imitation is so simple, why do non-human primates not simply copy what they perceive? The received view has formulated two responses.

Two decades ago it was still widely accepted that chimpanzees imitate less than humans because they lack the required social cognitive processes. For chimpanzees “the intentional states of the demonstrator [are] either not perceived or irrelevant,” whereas for humans, “the goal or intention of the demonstrator is a central part of what they perceive” (Tomasello, 1996, p. 331)^3^. This initial theory had to be revised after experimental evidence showed that apes understand that others have goals and behave toward them according to what they perceive. Instead it was claimed that apes still lacked an understanding of the “more mental dimensions of intentional action […] – specifically those that have to do with the decision-making process by which the actor generates action plans and, based on a rational assessment of reality, chooses one to enact in intentional action” (Tomasello et al., 2005, p. 685). However, this theory also had to be rejected because of growing evidence that chimpanzees have a range of social skills, including an understanding of others’ goals (Tomasello et al., 2003; Call and Tomasello, 2008). In particular, there is evidence for rational imitation in enculturated chimpanzees, i.e., the fact that chimpanzees are more prone to imitate those aspects of observed action sequences that appear to be intentionally made but whose causal reasons are not self-evident (Buttelmann et al., 2007).

The current verdict is therefore that “[a]pes understand that others have goals and perceptions and how these relate to one another in intentional action, perhaps even rational action” (Tomasello, 2008, p. 177). All of this undermines the original hypothesis that the propensity to imitate is positively correlated with an understanding of other minds. Indeed, this should not come as a surprise since even adult humans – presumably having the most sophisticated social skills of all animals – imitate significantly less than human infants (Horowitz, 2003). It therefore seems that other factors must be in play.

This leads us to the second response to this dilemma, which accepts that non-human primates have intentional understanding and that imitation should be more common among non-human primates. For example, “mirror” neurons were first discovered in macaques, and a widely accepted interpretation of their function holds that intentional understanding is based primarily on a mechanism that directly matches the sensory representation of the observed actions with one’s own motor representation of those same actions (Rizzolatti and Sinigaglia, 2007). Bodily mirroring can also be an emergent outcome of the coordination dynamics of social interaction (Froese et al., 2012b). Given that such “motor mimicry” is assumed to be an automatic response, and given that it is assumed that perception delivers the requisite physical details, it is surprising that monkeys (and primates in general) do not imitate each other all the time. Consequently, some researchers have turned their interests toward explaining the neural mechanisms of the active *inhibition* and control of, rather than *initiation* of, imitation (e.g., Brass et al., 2009; Rumiati et al., 2009). According to these researchers an individual requires intentional control to ensure that their imitation is goal-directed rather than compulsive (Heyes, 2009). Evidence of deferred imitation in enculturated chimpanzees supports the claim that imitation is not a mere reflex response, but rather an action that is under intentional control (Bjorklund et al., 2002).

But this response simply brings us to another version of the same conundrum: why do apes not *disinhibit *imitation more often, especially if they apparently have sufficient intentional control to *inhibit* its execution unless that imitative action suits their goals? Following this response, the empirical data is still rather puzzling because it seems to indicate that non-human primates, for no apparent reason, reliably fail to disinhibit their existing automatic imitative responses even when their intentionally directed emulation consistently fails to bring about the desired ends. For example, one study of captive chimpanzees demonstrated that they will continue begging for food in their usual, but evidently unsuccessful, manner without trying to copy the successful begging gestures of their specially trained conspecifics (Tomasello et al., 1997). We will discuss this negative finding in more detail later on.

And if non-human primates in general seem to exhibit too much active inhibition of imitation, then why do human children show too little of it, as suggested by their “surprisingly unselective ‘over-imitation”’ (Whiten et al., 2009, p. 2417)? For example, surely children have good enough physical cognition to quickly learn when turning a pin is in fact a non-functional motion, so why repeat that observed motion over a hundred times (Whiten et al., 1996)? Equally surprising, from this theoretical point of view, is the fact that imitation by adults is more like that of chimpanzees. Lieven and Stoll (2013) reported imitation in only the youngest children of their two-culture sample. Similarly, Horowitz (2003) found that human adults, like chimpanzees on a similar task, were significantly more likely to emulate than to precisely imitate actions that were demonstrated in the opening of an “artificial fruit.” And this happened even though the participants later claimed that they had believed themselves to be imitating all along. If they had indeed intentionally tried to disinhibit their automatic imitative response, why did they end up emulating the demonstration?

This tension between (a) the *a priori* assumption that perceptual experience provides a detailed mental representation of the physical environment, including of the motions of other agents, and (b) the empirical prevalence of emulation over imitation, warrants a reconsideration of the general validity of the HPP. Maybe mental states are not as perceptually hidden, and physical details not as perceptually evident, as has been hitherto assumed in comparative psychology.

## EVIDENCE FOR THE HDP FROM PHENOMENOLOGY AND PSYCHOLOGY

It may be argued that we cannot assess whether the HDP better accounts for the point of view of non-human primates because we cannot know what it is like for them to perceive the world. However, while we can never be absolutely certain about another agent’s first-person experience (Nagel, 1974), this kind of certainty is not required for doing science. It is still possible to motivate a more general acceptance of the HDP by realizing that it accurately describes our own point of view, and by demonstrating that its validity can be indirectly confirmed on the basis of its behavioral consequences.

### EVIDENCE FROM PHENOMENOLOGY

Phenomenologists have long emphasized that we normally experience ourselves to be embodied in meaningful situations (Heidegger, [1927] 1962; Merleau-Ponty, [1945] 2002). There is a consensus that under normal circumstances we directly perceive other persons as being intentional agents in their own right, and that much of others’ psychological states is immediately perceivable in the way in which they comport themselves in the world, especially in social interaction (Ratcliffe, 2007; Gallagher, 2008a; Zahavi, 2011). We cannot here review the vast literature of phenomenology as it pertains to other people (but see, e.g., Gallagher and Zahavi, 2008). Instead we provide an illustrative example of the phenomenological analysis of the perception of another person’s expressions. As Scheler ([1923]2008)) once remarked, although it may appear self-evident to “intellectualist” (i.e., cognitivist) theories that we perceive nothing of another person apart from their physical body and its objective movements in space, it only requires the simplest reflection about our own lived experience to show that there is nothing self-evident about this.

For we certainly believe ourselves to be *directly acquainted* with another person’s joy in his laughter, with his sorrow and pain in his tears, with his shame in his blushing, with his entreaty in his outstretched hands, with his love in his look of affection […]. If anyone tells me that this is not ‘perception’, for it cannot be so, in view of the fact that a perception is simply a ‘complex of physical sensations’, […] I would beg him to turn aside from such questionable theories and address himself to the phenomenological facts. (Scheler, [1923]2008, p. 260, emphasis added).

This insight about our direct perceptual experience of other people is not limited to the phenomenological tradition of philosophy. Similar descriptions can also be found in the analytic tradition, for example as famously expressed by Wittgenstein (see also, e.g., McDowell, 1982).

“We *see* emotion.” – As opposed to what? – We do not see facial contortions and *make the inference* that he is feeling joy, grief, boredom. We describe a face immediately as sad, radiant, bored, even when we are unable to give any other description of the features. – Grief, one would like to say, is personified in the face. (Wittgenstein, quoted in Overgaard, 2007, p. 128.)

The notion that the meaning of an expression or gesture can be directly perceived, while physical features are relatively inaccessible or absent, lies at the core of our hypothesis. Even a person’s whole physical individuality can be hidden behind their perceived meaning. For example, Gurwitsch ([1931]1979) has observed that other people are often primarily encountered in terms of their social roles, and that their role partially constitutes the meaning of a situation. He therefore remarks that individual role-bearers can be substituted for each other without much disruption to a social understanding of the situation, since “only in this role do I have something to do with him. In this situation, his being is exhausted in the role whose bearer he is” (Gurwitsch, [1931]1979, p. 108). Of course, other people only appear as completely defined by their social roles in some generic kinds of social situation, such as explaining directions to a stranger (as we will see in more detail below), handing your ticket to a train conductor, etc. More would need to be said about the ways in which others are experienced as individual people (Ratcliffe, 2007, pp. 58–84).

It is only during serious forms of psychopathology that the world and other people are experienced as nothing but a jumble of meaningless objects and mindless automata (Stanghellini, 2004). In such unfortunate cases the observer is forced to engage in explicit reflection and inference-making about the meaningless observed movements of others (thereby effectively creating a personal-level theory of mind^4^ in order to compensate for the lack of direct perceptual insight into the intentions and meanings of even the most basic kinds of actions (Froese et al., 2013). Indeed, accounts written by sufferers of schizophrenia give us phenomenological insight into what it could be like to only perceive the meaningless “surface” behavior of another person, as is assumed by the HPP. Consider the following description by a girl who lost her ability to directly perceive others’ embodied mindedness, such that she was confronted by an unbearable perception of another’s body as some kind of inanimate physical machine, rather than as an expressive body of another person in their own right:

I saw her eyes, her nose, her lips moving, heard her voice and understood what she said perfectly, yet I was in the presence of a stranger. […] She seems more a statue than ever, a manikin moved by a mechanism, talking like an automaton. It is horrible, inhuman, grotesque. (Renée, quoted in Sechehaye, 1970, pp. 36–38.)

This patient’s description makes it painfully clear that the HPP mischaracterizes our normal perceptual experience of other persons. We normally do not perceive others in terms of only their surface behavior, such as the mere movements of an automaton’s body parts; we normally directly perceive others as living, intentional agents just like ourselves, who act for reasons rather than merely mechanically – and we realize this without any extra need for cognizing.

### EVIDENCE FROM PSYCHOLOGY

Some scientists may find these phenomenological insights too anecdotal to be taken seriously, but there are a number of experimental paradigms in psychology that also support the HDP and that undermine the guiding assumption of the HPP, i.e., that we are normally presented with a highly detailed physical environment in our perceptual experience. We begin by highlighting evidence that the perceived meaning of an object partially hides its physical characteristics. We then discuss evidence that the perceived meaning of a situation can even hide whole objects and personal identities.

First, experiments in categorical perception have repeatedly demonstrated that our perceptual experience is shaped by a so-called “perceptual magnet effect,” which implies that the structures of our experience are partially constituted by our learned conceptual categories (Harnad, 2003). Certain physical details will be more or less accessible to experience depending on the categories by which we make sense of the world. Regarding social perception it has been demonstrated that our conceptual categories influence how we perceive others’ vocalizations (Iverson et al., 2003) as well as their facial expressions (Kotsoni et al., 2001). For example, discriminating others’ facial expressions within a meaningful category of emotion is more difficult than discriminating them across different categories, even if they differ by an equal physical amount (Etcoff and Magee, 1992). Specifically, given a computer-generated continuum of facial expressions from happy to sad, it is more difficult to tell apart two images of happy faces (or sad faces) than to differentiate between two images of faces that express an undefined feeling between happy and sad. Anticipation of emotions can also lead to the misperception of facial details (Palumbo and Jellema, 2013).

Systematic cultural differences in perception and social cognition have also long been reported by ethnographers (e.g., Lillard, 1998; Vinden, 1999; Boesch, 2007; Henrich et al., 2010). In psychology there is a field of study dedicated to elucidating how the natural and socio-cultural context of the perceiver shapes their experience, including their susceptibility to illusions (Caparos et al., 2012). There is also growing evidence that believing others to be intentional agents has top-down effects on perception, such as modulating how their physical movements are perceived (Moore et al., 2013) and on mechanisms of attentional selection (Wiese et al., 2012). The fact that there is a co-dependence between basic sensory processing of others’ physical characteristics and higher-level social understanding of others has been taken to support Wittgenstein’s observation that we experience ourselves as directly seeing other people’s emotions, intentions, and attention (Teufel et al., 2010).

**FIGURE 1:**
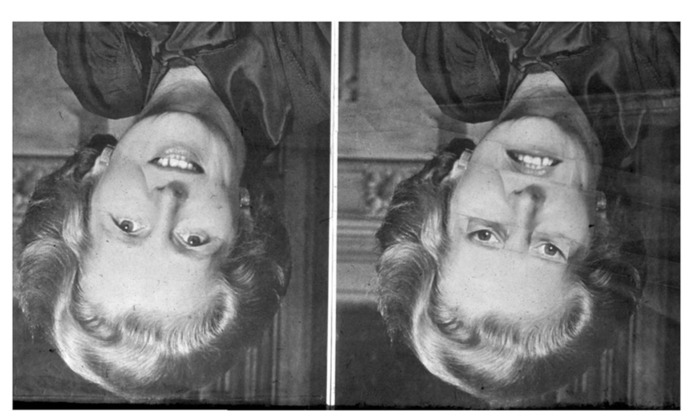
**The “Thatcher illusion ” (Thompson, 1980).** We first see two more-or-less identical faces. However, when they are turned around 180° to their proper orientation, it turns out that one face had been manipulated. These abnormal physical details had previously been perceptually obscured behind the meaningful experience of seeing a replication of the left-hand image. (This figure was first published in Thompson, P. “Margaret Thatcher: a new illusion” *Perception*, 1980, 9, pp. 483–484, reproduced by kind permission of Pion Ltd, London. Website: http://www.perceptionweb.com).

Since it may be difficult to intuitively grasp what it means to fail to notice physical details when perceiving another’s body, the reader is encouraged to experience this effect from her own first-person perspective. We therefore reproduce the “Thatcher illusion” (Thompson, 1980), which is particularly relevant for generalizing these kinds of findings to comparative psychology, because it has been demonstrated to apply to the perception of non-human primates as well, including chimpanzees and, to a lesser extent, monkeys (Nakata and Osada, 2012; Weldon et al., 2013). The perceiver sees two seemingly similar pictures of a smiling face when these pictures are positioned upside down, but not when they are turned to their upright position (**Figure 1**).

The effects of the Thatcher illusion are still relatively subtle, but “change blindness” (Simons and Rensink, 2005) and “in attentional blindness” (Mack, 2003) paradigms in psychology have provided extensive evidence that we often fail to notice substantial changes in a visual scene, such as the changing color of a car or the disappearance of a plane’s engines, even when asked to look for any changes taking place. Moreover, it appears to the participants that they perceive the scene as a whole without any factual gaps. While unusual orientations, flickering, splashes, and other artificial techniques help, they are not needed to induce these effects. Consider, for instance, the “gorillas in our midst” experiment (Simons and Chabris, 1999)^5^. Participants were instructed to count the number of basketball passes between members of one team of basketball players, all wearing the same-colored shirts. In the middle of the video a person dressed up in a full-body gorilla suit strolls right into the midst of the passing players. The “gorilla” stops to face the camera, pounds its chest, and then wanders off. Incredibly, around 50% of people fail to notice that anything out of the ordinary has taken place.

Researchers also found perceptual “blindness” in real-life social situations (Simons and Levin, 1998). They had an assistant pretend to be lost on campus and then to approach a random passerby for directions and for help in using a map. In the middle of this interaction two other assistants carrying a large opaque door rudely barged through the two interactants. During this brief interruption the “lost” person was quickly replaced with another person playing the same role, and afterwards the interaction continued. Astonishingly, in about 50% of cases the passerby failed to notice that their interlocutor had been swapped for a different person. In one variation of the experiment the two swapped people were both dressed as construction workers. The researchers comment: “One subject who failed to detect the change essentially stated our predicted hypothesis: She said that she had just seen a construction worker” (Simons and Levin, 1998, p. 648). They concluded that, provided that the overall meaning of the scene is unchanged, physical changes to seen persons often escape awareness even when they occur during a natural, real-world interaction.

These findings are surprising from the representationalist perspective of the HPP, but they are in accordance with the HDP. Absence of physical details is to be expected if experience is primarily about situating perceivers in a meaningful and goal-directed relationship with their environment, for example based on our capacities for action (Noë, 2002). And the same applies to our experience of other people. For example, the person-swapping experiments provide empirical support for the phenomenological analysis by Gurwitsch ([1931]1979) about how other people are often primarily perceived in terms of their social roles. Although more comparative research needs to be done in order to determine to what extent these findings can be generalized to the perceptual experience of non-human primates, there are no *a priori* reasons to assume that they are exempt from meaningful perception and its consequences. Moreover, as the next section demonstrates, such a generalization is theoretically supported because of the parsimonious account it provides for comparative research on imitation.

## LOGICAL CONSEQUENCES OF THE HDP REGARDING IMITATION

We are now in a position to take a closer look at the logical consequences of the HDP for current issues in imitation research.

HDP-(1): The intentional actions of other agents are primarily perceived in terms of their meaning and goal-directedness.
HDP-(2): Given HDP-(1), the other’s abstract physical motions in objective space are not primarily observed via perceptual experience.
HDP-(3): Given HDP-(2), observation of the other’s abstract physical motions in objective space requires additional cognitive effort.

Note that HDP-(2) is a less constraining consequence than HPP-(2), because even though direct perception is the normal default mode, perception of physical details is not necessarily impossible altogether. But the HDP predicts that perception of abstract physical details of meaningful actions requires additional cognitive effort of some kind, such as detached observation, controlled shifts of attention, explicit analysis of isolated perceptual stimuli, and so forth. This has logical implications for the way in which observed actions can be replicated.

HDP-(4): Given HDP-(2), an agent’s replication of the observed behavior of another agent is primarily guided by the other’s perceived goals.
HDP-(5): Given HDP-(3), an agent’s replication of the observed behavior of another agent can also be guided by the other’s perceived physical motions, but only to the extent that the observer has the required additional capacities.

We therefore arrive at the following prediction:

HDP-(6): Given HDP-(4) and HDP-(5), copying the means of an observed action is cognitively *more* demanding than emulating its intended results.

In other words, according to the HDP, because the physical details of an observed meaningful action tend to be obscured by top-down effects on perception, the default mode of replicating behavior is emulation. On this view, and in direct contrast to the traditional view of imitation (e.g., Tomasello, 1999), understanding the other’s goals and intentions does not facilitate imitation, but actually hinders it because such direct insight obscures the precise means. Imitation requires individuals to change attention from *what* the other’s goals are to *how *the other’s actions are precisely realized, while emulation is possible without this extra effort. This proposal better accords with the fact that faithful imitation is less common in non-human primates although they have a range of social capacities, including goal understanding (Tomasello, 2008, p. 177).

It could be countered that neonates lack the requisite cognitive skills to control their focus of attention, and yet they are still able to imitate with flexibility. However, the need for higher-level cognition to reveal the physical details of the means of an observed action is premised on the fact that the action has an intelligible goal. But if the other’s goal is completely unintelligible, for example because it is an unfamiliar conventional practice, then there is no perceived meaning that could be competing perceptually and attentionally with recognition of the fine-grained physical means of the other’s action. In other words, if the other’s goal is not directly perceptually given, then a replication of the observed behavior should be more easily guided by its actual physical execution – without any need for extra cognitive effort.

These considerations amount to the related hypothesis that an individual’s propensity to emulate is dependent on the direct intelligibility of the observed action. Conversely, imitation is expected to be more frequent whenever the goal of the other’s action is perceptually opaque. According to HDP, therefore, it is possible to evaluate the space of observable actions according to their potential direct perceptual intelligibility when viewed by a conspecific. We propose three broad partially overlapping categories that lead from intelligibility to opacity:

(1) Biologically constrained behavior (completely intelligible to conspecifics),
(2) Contextually constrained behavior (partially intelligible to conspecifics), and
(3) Conventionally constrained behavior (completely unintelligible, unless seen by an enculturated group member).

The category of “biologically constrained behavior” includes all communicative expressions and actions whose meaning and goals are constrained by species-specific biology. Understanding of such actions is instinctual. For example, a wolf will never misunderstand a conspecific wagging its tail. We predict that copying of this kind of behavior will nearly always be emulative, while precise imitation is exceedingly difficult because it requires a concerted effort of detachment and analysis (although in practice the limited number of alternative means to achieve the same result may make this replication appear close to copying the means).

The category of “contextually constrained behavior” includes more ambiguous action types, because intelligibility also depends on the relation of the observed behavior to the observed social and natural context. The more species-atypical and mediated the action is, the less intelligible it appears. For example, when one ape observes another one reaching into a tree, grabbing something and then putting it into its mouth, this should be directly perceived as feeding without need for reflection. Intelligibility of this behavior is not simply determined by species-specific biology, but rather enabled by the characteristics of the situation, i.e., by directly observing the effects that an action has on the environment. On the other hand, the paradigmatic class of more mediated behaviors is the learned use of tools, for example when a chimpanzee uses a stick to fish for insects or honey (Humle et al., 2009). The way in which a behavior of this category will be copied depends on how self-evident its function is. The default mode of copying will still be emulation. However, aspects of instrumental action and tool-use whose intentions are obscured, including conventionally determined norms of behavior, require closer observation and more imitative learning, thereby leading to the emergence and preservation of cultural traditions that are not determined by functional considerations alone. While most pronounced in humans, such traditions have also been demonstrated in non-human primates, including chimpanzees (Whiten et al., 1999), orangutans (Krützen et al., 2011), and capuchin monkeys (Perry, 2011).

Uniquely, the perceived meaning of a “conventionally constrained behavior” is neither constrained by an internal biological necessity nor by the external environment. Instead, the relationship between a behavior and its meaning is primarily based on a social convention that is largely arbitrary. The reasons for the specific form of the relationship are irrelevant and usually not known to the community. Although not absent in non-human primates, this category of behaviors is especially typical for human actions, including language, customs, and rituals. More recently, it also includes writing and complex technology. In these cases the default mode of copying an observed behavior will be imitation, at least for young children and other cultural outsiders, because its meaning is not directly intelligible without having already been enculturated. Our theory therefore predicts that imitation is most frequently done by young individuals in response to the perception of unfamiliar social behavior while developing in a richly symbolic culture.

We also predict that the more an action is conventionally constrained, the more it will be faithfully imitated by group members, thereby restricting variation in performances of that action within the community. For example, in the case of chimpanzees we therefore expect there to be more variation in using a stick to fish for termites (a mostly contextually constrained action) in contrast to the hand–clasp interaction during mutual grooming (a mostly conventionally determined action). However, this prediction is also age-dependent. During enculturation imitation is eventually replaced by emulation, because as the meaning of conventionally constrained actions is learned, their meaning will become perceptually transparent just at the same time as the perception of their underlying physical means will require increasing effort. As we know in the case of humans, the perception of certain physical details will eventually become nearly impossible for adults, for example the distinction between the sounds of the English “l” and “r” by adult Japanese speakers.

Regarding non-human primates there is a classic experiment conducted by Tomasello et al. (1997), which investigated imitation in response to a novel arbitrary social gesture that did not involve tool-use in two groups of captive chimpanzees. Since the outcome of this experiment at first sight appears to contradict our theory, we will consider it in more detail here. On three occasions the experimenters temporarily separated a dominant female chimpanzee from her group to extensively train her alone to perform an arbitrary gesture to receive treats. After the trained chimpanzees were returned to their groups, they were called to the fence where they spontaneously began to perform the learned gesture, and thus received the coveted treats in full view of the other members of their group. The other members were highly motivated to get their own treats, but they performed their usual begging gestures to the experimenters; none of them attempted to imitate the new gestures of the trained chimpanzees.

However, worries have already been raised about the possibility that negative findings derived from experimental paradigms using food-related actions might fail to generalize to the imitation of social conventions (Watson and Caldwell, 2009). Relatedly, from the perspective of our theory, the main problem with this particular study is that it tried to replace an existing instinctive gesture with a novel gesture within a highly familiar context, i.e., begging from humans. The highly familiar situation of food-procurement enables chimpanzees to directly understand a conspecific’s begging action as such in a contextually enabled manner, no matter that it is performed differently. This social understanding of the other’s goal, according to our theory, makes emulation the more likely response. Moreover, there already existed an instinctual response, the open-hand begging gesture, which is universally understood by humans and chimpanzees alike. Indeed, the experimenters recorded these normal begging gestures, showing that there was no communication problem as such. The chimpanzees understood what the trained chimpanzees were doing, i.e., begging for food, and they also understood that the experimenters understood what they themselves were doing, i.e., begging as well. Given this general understanding of the situation by the observing chimpanzees, our theory predicts correctly that the physical details of the trained gestures were obscured behind a direct perception of the other’s goals, thereby leading to emulation.

Note that the two theories of perception make differing predictions regarding the role of perception and intelligibility for imitation. First, whereas the HPP entails that the understanding of a perceived action is a secondary, independent cognitive process, the HDP entails that understanding cannot be easily separated from perception itself. As we have shown, there is a growing body of evidence to support the latter assertion. In terms of measurable effects on imitation, there is at least one study of that directly manipulated perceptual experience, and the results are better accommodated by the HDP. It was found that perceptual salience of the demonstrated behavior, as varied by familiarization and distractors, modulates infants’ propensity for rational imitation (Beisert et al., 2012). For instance, when an adult operated a light switch using their head while their hands were visibly unoccupied, 10 out of 14 (73.3%) infants imitated the action, which seems like a rational choice because there could have been an important reason for avoiding the easier option of using the hands. But when the experiment was repeated with two distracting smiley faces placed on the table, one next to each of the unoccupied hands, the tendency for such “rational imitation” was attenuated: although the rational choice should have been the same as before, only 8 out of 15 infants imitated the action (53.3%; Beisert et al., 2012, p. 3). It is therefore possible that some negative results of imitation in non-human primates also suffered from the perceptual effects of distraction and lack of salience.

Second, whereas the HPP entails that the physical details of the world are just as easily perceived no matter their intelligibility, because intelligibility is a later stage of inferential processing, the HDP entails that physical details and intelligibility are in conflict with each other. To be sure, both the HPP and the HDP are compatible with findings showing that diminished intelligibility is positively correlated with imitation. But whereas the HDP leads us to the prediction that imitative responses become less frequent with increased intelligibility, the HPP is neutral on this point or even predicts the opposite, e.g., the traditional hypothesis that humans have a greater ability for imitation compared to chimpanzees because humans are better at making sense of others’ goals (i.e., only they have a “theory of mind”). It is therefore also possible to arbitrate between these theories by evaluating the extent to which the absence of imitation is related to the presence of intelligibility.

## THEORIES OF THE DEVELOPMENT AND EVOLUTION OF IMITATION

We finish by contrasting the consequences of the HPP and HDP in terms of theories of the development and evolution of imitation.

### EXPLAINING THE DEVELOPMENT OF IMITATION

Imitation starts from the first moments of life. For example, it has been found that human neonates can imitate a variety of arbitrary facial gestures (Meltzoff and Moore, 1977), but so can chimpanzee neonates (Myowa-Yamakoshi, 2006; Bard, 2007) and even macaque neonates (Ferrari et al., 2006). Delayed imitation studies with 1-week-old rhesus macaques suggest that these are not mere reflexes, but are to some extent under intentional control (Paukner et al., 2011). This prevalence of early imitation is what we would predict given that young infants do not yet have well-developed social understanding of even the most basic gestures, and are thus more likely to perceive physical features. Conversely, it is expected that this precise imitation decreases as basic social competence increases, because the development of categorical perception and social understanding will start to obscure the physical details of what is directly perceived as meaningful. And, indeed, it is well known that human neonatal imitation disappears after 2–3 months of age (Jones, 2009), and a similar developmental trend exists for chimpanzee neonates:

At less than 7 days of age, the chimpanzees could discriminate between and imitate several human facial gestures. However, by the time they were 2 months old, the chimpanzees no longer imitated the gestures. They began to perform the mouth pen (MO) gesture frequently in response to any of the three facial gestures presented to them. This response could be considered as “social smiling” (i.e., play face) directed at the human experimenter (Myowa-Yamakoshi, 2006, pp. 223–224).

Myowa-Yamakoshi (2006) also noted that the disappearance of facial imitation might be related to social-interactive responses toward the experimenter. We agree and suggest that the physical realization of the facial gesture, i.e., face with/without tongue and/or lip protrusion, has become perceptually obscured by the acquired basic social understanding. The otherwise puzzling disappearance of neonatal imitation in human and non-human primates can therefore be understood in terms of the development of a basic, still largely biologically constrained, social competence.

Young humans’ propensities for faithful imitation of arbitrary gestures reliably reappear after 1.5–2 years of age (Jones, 2009). Infants’ over-imitation of tool-related actions, even of evidently unsuccessful ones, increases with age, becoming the default response after around 2 years of age (Nielsen, 2006). This reappearance of imitation could reflect a new social learning process that specifically responds to the unintelligibility of conventionally constrained behavior, such as culturally mediated social interaction. Relatedly, we expect that emulation will once again become the default mode of imitation in adults, because most conventionally constrained actions will by then have become perceptually intelligible, thereby once again obscuring the underlying means. Indeed, in Horowitz’s (2003) study of adult human imitation the details of the demonstrator’s motions apparently remained outside of the observer’s perceptual focus, as confirmed by anecdotal reports: “In casual conversation during the debriefing period, 1 subject remarked after hearing that the experiment gauged her level of imitation ‘Oh, you mean when I saw you messing with the box, if I imitate *that*?’ ” (Horowitz, 2003, p. 333). We suggest that the participants primarily saw the demonstration as a general “messing about” whose physical details were perceptually obscured by the self-evident goal of opening the device.

Interestingly, a similar later developmental trend has been observed in the case of chimpanzees. After the disappearance of neonatal imitation, a reemergence of precise imitation has been observed to occur around 9 months of age (Myowa-Yamakoshi, 2006). In one field study several young chimpanzees, but none of the adults, were documented to imitate the idiosyncratic actions of a disabled adult chimpanzee (Hobaiter and Byrne, 2010). Thus, at some point the propensity for imitation in young chimpanzees decreases once again, as is also demonstrated by a host of experiments involving captive adult chimpanzees (e.g., Tomasello et al., 1987, 1997; Nagell et al., 1993; Bjorklund et al., 2002). This broad similarity to the non-linear development of imitation in young humans suggests that juvenile chimpanzees may also aim to acquire the conventionally determined behavior of their group.

### EXPLAINING THE EVOLUTION OF IMITATION

One popular hypothesis is that human imitation first emerged because of a necessity for young individuals to learn complex tool-making techniques (Csibra and Gergely, 2006). The main idea is that humans are more prone to imitation because natural selection honed them to focus their attention on others’ complex tool-related actions, rather than just their goals or effects on the environment (Tomasello, 2008, pp. 208–209). At the same time it is recognized that the success of imitative learning depends not so much on slavishly copying the others’ movements, but also on a hierarchical analysis of overall goals and plans leading to “program-level” imitation (see also, e.g., Byrne and Russon, 1998; Tomasello et al., 2005). On this view, faithful imitation was only later adapted for imitating socially determined behavior (Tomasello et al., 2005, p. 687).

We agree that successful imitation depends on learning to refocus attention to specific aspects of observed actions, although our account differs slightly. Evidence for so-called “program-level imitation” (Byrne, 2003) fits with the idea that observers first perceive the other’s general intention, while refocusing on the physical details of the component movements requires additional effort. Nevertheless, the hypothesis that precise imitation in humans evolved specifically because of the need to copy complex tool-use does not sit easily with the experimental evidence. Over-imitation by children and under-imitation by adults are puzzling phenomena if precise copying of tool-based functionality was the primary evolutionary pressure for human imitation.

There is another issue with the hypothesis of tool-related origins of imitation, which is the tendency of overestimating the opacity of observed tool-use behavior. Apart from complex modern technology, most learning of new tool-use practices can be guided by close observation and practice, as demonstrated by young chimpanzees in the wild (Inoue-Nakamura and Matsuzawa, 1997; Biro et al., 2006). However, no matter how many times you say “bring me that ball” to a pre-linguistic infant, the meaning of this communicative action will remain elusive unless it is tied into a pragmatic context involving the speaker, the listener, and a ball (Tomasello et al., 1993). We therefore agree with Csibra and Gergely (2009) that the acquisition of human cultural practices is facilitated by specialized gestures, such as faithful imitation and ostensive signals. However, on our view, they misjudge what precisely is special about human culture by accepting the HPP as their starting point.

[T]o acquire the relevant knowledge through observation sets an ill-posed *inverse problem*: a behavior can always be generated and explained by an infinite number of different mental state combinations, representing diverse goals and/or different types of background knowledge. This difficulty is just multiplied when observing mediated (recursive) tool use [e.g. when one tool is used to produce another tool], where no perceptible reward would inform the observer about the tool’s function and, in the absence of that, there is no way to assess the relevance of any element of the behavior observed. (Csibra and Gergely, 2006, p. 252.)

The HPP commits Csibra and Gergely to the questionable claim that, from the point of view of an external observer, any behavior could be caused by an “infinite” number of mental states because nothing but the physical states of an action are observable. However, according to the HDP, such an absolute “inverse problem” normally does not exist in practice, even when observing recursive tool-use. Csibra and Gergely illustrate the concept of recursive tool-use by contrasting a child’s observation of someone using a tool to peel away the hard skin of a fruit (presumably to eat its interior) compared to the child observing someone using a tool to carve away bits of a piece of wood (presumably to make a pointy spear). We agree that the latter, recursive action would be less intelligible than the former, but many of its aspects would still be sufficiently contextually constrained to be intelligible for the child. For example, the wood carver’s attention will be focused on the shape of the tip (and not on the flakes falling down or the sounds that are made); he may look at it, feel it with his fingers, clean away bits that get stuck, etc. He may also throw the spear at some target to check its effectiveness, and if not satisfied, continue carving some more. Once done, he will take the resulting spear on the hunt where its utility in killing prey will be put to the test; if it happens to break, he may carve a new tip. In other words, the meaning of the tool-based making of this tool is largely intelligible because it is embedded in contextually constrained practices.

To be sure, Csibra and Gergely’s general hypothesis that imitation is related to the copying of unintelligible behaviors matches our own theory (see also Gergely and Csibra, 2006), but their commitment to the HPP prevents them from appreciating the qualitative difference between observing tool-use (even of the recursive kind) and observing actions that are based entirely on social conventions. We argue that it is only when the child is observing an unfamiliar conventionally constrained behavior that she is in fact confronted by genuine opacity. This is why over-imitation, as well as pedagogy (Csibra, 2007), are observed mainly in humans: our survival and success depends on learning social conventions. Young non-human primates are also keen to learn tool-based actions from adults and exhibit some imitative learning. For example, young chimpanzees engage in long periods of observational learning of cracking nuts by using an anvil and hammer stone (Biro et al., 2006), and young capuchin monkeys have also shown to exhibit observational learning of tool-use (Fredman and Whiten, 2008). But, as far as we know, young non-human primates in the wild, in the absence of extensive symbolically mediated social conventions like those characterizing humans, have to learn only a limited number of arbitrary social conventions in order to become successful group members, and the opacity of others’ actions is therefore more manageable.

The unique perceptual opacity of unfamiliar conventionally constrained behavior also helps to clarify the relationship between imitation and social conformity. It has been hypothesized that the higher prevalence of imitation in humans could derive from their need to enact an appropriate sociocultural identity so as to become an accepted member of their social group. On this view, human imitation is premised on a shared social contract, whereas “there is no good evidence that apes imitate others only for social conformity and/or solidarity” (Tomasello, 2008, p. 213). We agree with the idea that imitation helps young humans to develop into successful members of their group, but we argue that this phenomenon is not strictly limited to humans.

For example, an experiment with captive chimpanzees has found that adults tend to conform to norms of tool-use even if other styles of usage are known (Whiten et al., 2005a). That this social conformity is partially related to affirming group membership cannot be ruled out. For instance, chimpanzee neonates are more likely to imitate in a communicative situation (Bard, 2007), and young chimpanzees rarely imitate facial gestures in the absence of ongoing bodily contact with the demonstrator, which suggests that social bonding is an essential element of their imitation (Myowa-Yamakoshi, 2006). Solidarity is also observed. For example, adult chimpanzees help each other upon request even when there is no immediate possibility for reciprocity (Yamamoto et al., 2009), and they console victims of bullying (Fraser et al., 2008). Adult bonobos collaboratively share food in the wild (Savage-Rumbaugh et al., 1998, pp. 219–225). These findings are not restricted to apes. Conformity to social norms of food preference is documented for wild vervet monkeys (van der Waal et al., 2013), and imitation has been shown to enhance social bonding in capuchin monkeys (Paukner et al., 2009). However, despite this growing evidence of social conformity and solidarity, there is little evidence of over-imitation in non-human primates.

These considerations lead us to formulate a revised hypothesis regarding the origin of imitation in humans. We agree with Tomasello (2008) that the greater prevalence of imitation in young humans is related to the formation of a shared social contract, but we specify that this is because the human social contract is defined by conventionally constrained practices. Our social identities are largely constituted by the arbitrary symbol systems in which we grow up. From this perspective it is no longer surprising that human children are especially prone to over-imitation. Their best bet of becoming enculturated into their social group is by focusing their imitative learning on opaque actions whose function does not appear contextually constrained. Over-imitation is therefore far from being surprisingly unselective. It is a highly selective behavior that picks out precisely those aspects of social interaction that are most likely part of conventionally constrained practices. In other words, over-imitation is an effective developmental response to becoming enculturated in a symbolic culture. Similarly, enculturated apes – apes who were cross-fostered by humans and who have been embedded in richly symbolic environments – imitate arbitrary actions more readily than do other institutionalized apes (Custance et al., 1995; Rumbaugh et al., 2008).

## CONCLUDING REMARKS

All theories of imitation agree that the more conventionalized social interactions are, the more imitative social learning is required to become a successful member of the group. However, theories based on the HPP and our theory based on the HDP differ in one crucial respect: the former are united by the assumptions that physical details are always and exclusively perceived, and that this perceptual access is independent of any subsequently realized intelligibility. Our proposal proceeds on experimental and phenomenological insights that the direct perception of an action’s goal and meaning is primary, and that there is therefore a conflict between perception of physical details and their intelligibility. Perception and intelligibility are joined together in a process of sense-making that accords primacy to meaning over physical details whenever this is possible, and if not counteracted by extra cognitive effort. Replacing the HPP with the HDP leads us to make several predictions that better fit the data.

We expect that an individual developing in a highly conventional cultural context will be more prone to faithfully imitate, as most theories of imitation do; but we explain this insight more consistently than other theories by emphasizing that conventionally constrained behaviors are uniquely opaque to young and uninitiated observers when compared to other types of action. We go against existing theories by predicting that an individual’s propensity to imitate is inversely correlated with her development and enculturation, as indexed for example by age and social competence. Rather than facilitating imitation, the acquisition of social understanding grants the observer direct perceptual intelligibility of others’ behaviors, such that emulation will ultimately become the default mode of copying behavior in adulthood. Faithful imitation, on the other hand, will increasingly require additional effort because the underlying physical details of others’ expressions will become perceptually obscured behind their directly understood significance. The proposed inverse correlation between direct perception and faithful imitation also explains why emulation is comparatively more frequent in non-human primates, since most of their behaviors are instinctively and contextually intelligible while only a few behaviors are conventionally constrained. In this way the HDP is able to explain a wider range of data in a more parsimonious manner.

## AUTHOR CONTRIBUTIONS

Tom Froese formulated the hypothesis and wrote the first draft of the manuscript. Tom Froese and David A. Leavens improved the text over several iterations.

## Conflict of Interest Statement

The authors declare that the research was conducted in the absence of any commercial or financial relationships that could be construed as a potential conflict of interest.

## Footnotes

1 There may be other ways of defending the phenomenological insights of the HDP apart from the HEC, for example in terms of subpersonal versions of Simulation Theory (Gallese, 2005) and Theory Theory (Gopnik, 1993). But what is more important here is that defenders of these competing theories are nevertheless in general agreement that the HDP makes a valid description of our personal-level social phenomenology (Spaulding, 2010).

2 We use the term “perceptual experience” rather than the more common “perception”, because the latter is sometimes interpreted as referring to a subpersonal process, while we are specifically interested in describing the subject’s own first-person perspective on the world. Although we sometimes use the term “perception” out of convenience, it should always be understood in this personal-level sense.

3 Tomasello here seems to explicitly acknowledge that humans can directly *perceive* the goals and intentions of others, as proposed by the HDP. However, to our knowledge he does not further develop the implications of this phenomenological insight in his theories.

4 Mainstream opinion holds that many forms of psychopathology are caused by an absence or malfunction of “Theory of Mind”, whereby the latter is defined as the process of making of inferences about other people’s states of mind on the basis of their physical movements. However, the reverse seems to be more correct, i.e., that because these psychopathologies involve a disruption of our normal direct perceptual insight into others’ meaningful expressions, patients are confronted with nothing but others’ physical movements and must therefore theorize about their hidden meanings – with limited success (Froese etal., 2013).

5 The video is available here: http://www.youtube.com/watch?v=vJG698U2Mvo
